# Prevalence of Childhood Obesity by Country, Family Socio-Demographics, and Parental Obesity in Europe: The Feel4Diabetes Study

**DOI:** 10.3390/nu14091830

**Published:** 2022-04-27

**Authors:** George Moschonis, George Siopis, Costas Anastasiou, Violeta Iotova, Tanya Stefanova, Roumyana Dimova, Imre Rurik, Anette Si Radó, Greet Cardon, Marieke De Craemer, Jaana Lindström, Luis A. Moreno, Pilar De Miguel-Etayo, Konstantinos Makrilakis, Stavros Liatis, Yannis Manios, on behalf of the Feel4Diabetes-study Group

**Affiliations:** 1 Department of Food, Nutrition and Dietetics, School of Allied Health, Human Services and Sport, La Trobe University, Melbourne 3086, Australia; g.moschonis@latrobe.edu.au (G.M.); G.Siopis@latrobe.edu.au (G.S.); 2 Department of Nutrition and Dietetics, School of Health Science and Education, Harokopio University, 17671 Athens, Greece; acostas@hua.gr; 3 Department of Endocrinology, Medical University Varna, 9002 Varna, Bulgaria; iotova_v@yahoo.com (V.I.); t92@abv.bg (T.S.); dr.roumyana.dimova@gmail.com (R.D.); 4 Hungarian Society of Nutrition, 1088 Budapest, Hungary; rurik.imre@med.unideb.hu (I.R.); rado.sandorne@foh.unideb.hu (A.S.R.); 5 Department of Movement and Sports Sciences, Faculty of Medicine and Health Sciences, Ghent University, 9000 Ghent, Belgium; Greet.Cardon@UGent.be; 6 Department of Rehabilitation Sciences, Ghent University, 9000 Ghent, Belgium; Marieke.DeCraemer@UGent.be; 7 Research Foundation, Flanders, 1000 Brussels, Belgium; 8 Department of Public Health Solutions, Finnish Institute for Health and Welfare, 00271 Helsinki, Finland; jaana.lindstrom@thl.fi; 9 Growth, Exercise, Nutrition and Development (GENUD) Research Group, Instituto Agroalimentario de Aragón (IA2), Instituto de Investigación Sanitaria de Aragón (IIS Aragón), Universidad de Zaragoza, 50009 Zaragoza, Spain; lmoreno@unizar.es (L.A.M.); pilardm@unizar.es (P.D.M.-E.); 10 School of Medicine, National and Kapodistrian University of Athens, 11527 Athens, Greece; kmakrila@med.uoa.gr (K.M.); s.liatis@yahoo.com (S.L.)

**Keywords:** BMI, childhood obesity, community intervention, Feel4Diabetes, lifestyle intervention, overweight, prevalence, prevention, school, socio-economic, type 2 diabetes, weight

## Abstract

The Feel4Diabetes study recruited 12,193 children (age: 8.20 ±1.01 years) and their parents from six European countries as part of the broader attempt to prevent type 2 diabetes. The current work collected data pre-intervention to identify the prevalence of childhood obesity by country and describe its association with socio-demographic characteristics and parental obesity status. One in four children were overweight or obese, and one in four families had at least one obese parent. Multivariate logistic regression examined the associations between childhood obesity, family socio-demographics, and parental obesity status. Children had a higher chance of being overweight or obese if they were living in “low income” countries (OR: 2.11, 95% CI: 1.62, 2.74) and countries “under economic crisis” (OR: 2.48, 95% CI: 1.89, 3.24) compared to “high-income” countries; if their fathers completed fewer than nine years of education (OR: 2.16, 95% CI: 1.54, 3.05) compared to children whose fathers had a higher level (>14 years) of education; and if one (OR: 2.46, 95% CI: 0.32, 0.62) or both of their parents (OR: 6.83, 95% CI: 5.15, 9.05) were obese. Future childhood obesity prevention-programs should target the whole family while taking into consideration the socioeconomic and weight status of parents. Future research should examine these associations in more countries and in socio-demographically diverse populations in order to facilitate the generalisability of the present study’s findings.

## 1. Introduction

Overweight and obesity affect more than one-third of the global population, with more than 1.9 billion adults and nearly four hundred million children and adolescents being overweight or obese according to relevant data reported by the World Health Organisation (WHO) in 2016 [1]. Overweight and obesity are well known risk factors for non-communicable diseases such as cardiovascular disease, type 2 diabetes (T2D), and certain forms of cancer, and collectively account for more than forty million deaths globally each year [2]. Overweight and obesity have been recently recognised as a risk factor for coronavirus disease-19 (COVID-19), with the Centers for Control and Disease Prevention (CDC) in the US reporting that four in five people hospitalised in the US with COVID-19 being overweight or obese [3]. The World Obesity Federation emphasised a “dramatic correlation” between COVID-19 deaths and obesity rates in “the 2021 atlas on COVID-19 and obesity” [4].

Obesity is increasing rapidly, with worldwide prevalence having nearly tripled in the thirty years between 1975 and 2016 [1]. More alarmingly for the future, childhood overweight and obesity rates are increasing at an even faster rate, with the prevalence of overweight and obesity among children and adolescents aged 5–19 years, having risen from 4% in 1975 to more than 18% in 2016 [1]. The obesity trend is even steeper than that of being overweight in children and adolescents, with 6% of girls and 8% of boys being categorised as obese in 2016, compared to less than 1% in 1975 [1].

Previous research has highlighted the link between obesity trends and social connections [5]. Similar to communicable diseases that can be passed on through physical infections, it appears that obesity can be spread through social networks, with chances of becoming obese increasing by 57% for people who have an obese friend [5]. However, it is not well established how parental sociodemographics, weight status, and the family environment influence the prevalence of childhood obesity and relevant trends.

Importantly, this once considered a high-income country problem, is now widely prevalent in both low- and middle-income countries [1]. However, obesity prevalence is disproportionate among population groups of the same country. Although it has traditionally been reported that certain groups with low socio-economic status (SES) are at a higher risk for obesity [6], new analysis from the WHO European Childhood Obesity Surveillance Initiative (COSI) indicates that although in the higher-income countries of the region there are lower obesity prevalence rates among people with higher SES, the trend is the opposite in countries with emerging economies [7]. The most current COSI data disprove the popular idea that low SES is consistently associated with less healthy behaviours and diets. This fact emphasizes the need to identify the socio-economic and other environmental correlates of childhood obesity in countries with different socio-economic classifications, thus informing country-specific public health policy. In this regard, better understanding of the factors associated with childhood obesity is an important preliminary step for tailoring effective interventions to tackle this ever-growing problem.

This study aimed to shed light on this area of research by using pre-intervention socio-demographic and anthropometric data collected from children and their parents participating in the Feel4Diabetes study [8]. Feel4Diabetes was a multicentre study with the primary aim of assessing the effectiveness of school- and community-based intervention in promoting healthy lifestyles, tackling obesity, and preventing T2D among families from vulnerable population groups in Europe [8,9,10]. Another important aim of the Feel4Diabetes study was to identify and report the correlates of energy balance-related behaviours and excess weight status of children and adults participating in the study. In this context, the objective of the current work was to report the prevalence of childhood obesity in the participating European countries, namely, Belgium, Bulgaria, Finland, Greece, Hungary, and Spain, and to examine any potential associations with the socio-demographic characteristics and obesity status of the parents.

## 2. Materials and Methods

### 2.1. Study Design and Sampling Procedures

The Feel4Diabetes study (http://feel4diabetes-study.eu/ (accessed on 24 March 2022), NCT02393872) was a large-scale community-based, family-involved study that aimed to promote a healthy lifestyle, including healthy eating and increased physical activity, in families from six European countries (Belgium, Bulgaria, Finland, Greece, Hungary. and Spain). The implementation of the Feel4Diabetes intervention took place over a total duration of two years (2016–2018).

The study was conducted within selected provinces of the participating European countries; recruitment was based on a standardised, multi-stage sampling procedure [8]. Specifically, in Bulgaria (an Upper Middle Income Country or UMIC according to The World Bank Country and Lending Groups classification [11]), all the municipalities within the participating regions were eligible for recruitment, while in Belgium, Finland, Greece, Hungary, and Spain (High Income Countries, or HICs) families within low SES municipalities were recruited. In HICs, low SES municipalities were defined as those with the lowest level of education and/or the highest unemployment rate as retrieved from official resources and local authorities within each country.

The details of the study design have already been published [8]. In brief, primary schools in the selected municipalities served as entry points to communities. Parents of children in the first three grades of these schools were invited to participate in the study.

### 2.2. Ethics Approvals and Consent Forms

The Feel4Diabetes study adhered to the Declaration of Helsinki and the conventions of the Council of Europe on human rights and biomedicine [12]. Prior to initiating the study, researchers in participating countries obtained ethics approval from local authorities. Participants were presented with a detailed description of the study and asked to fill in and sign consent forms for their participation, while they were also given the chance to withdraw from the study at any point.

### 2.3. Data Collection

Data were collected by trained researchers at baseline (2016) and during the first (2017) and second (2018) years of the program [13].

#### 2.3.1. Socio-Demographic Characteristics

Demographic and socio-economic information (e.g., age, gender, race, education, marital and employment status) was collected via self-reported questionnaires. More specifically, both parents reported their exact birth dates, which were then used to calculate their age. The age of parents was then dichotomized into <45 years vs. age ≥45 years. Forty-five years is generally considered the age at which middle age starts [14,15], although the exact age boundary is disputed [16]. This phase is marked by gradual physical, cognitive, and social changes in the individual. Regarding parental educational level, both parents were asked to report their “highest level of education completed” and choose one of the following portions: 6 years or less; 7–9 years; 10–12 years; 13–14 years; 15–16 years; and >16 years. Father’s and mother’s education were then grouped into three categories, <9 years, 9–14 years and >14 years, also on the basis thatthe duration of mandatory education in most European education systems is nine years [17]. Regarding race/ethnicity, parents that filled out the questionnaire had to respond to the question “To which racial or ethnic group(s) do you most identify?” by choosing one of the following answer options: “Caucasian/White; Black; Asian/Pacific Islanders; Latino or Hispanic; Chinese, Japanese, or other South-East Asian; Arabic or North African; Mixed; Other/Please specify.” In terms of marital status, parents answered the question “What is your marital status?” by choosing one of the following: “single, married or cohabiting; separated or divorced; widowed; other.” Finally, data on parental employment status were collected via the question “What is your main occupation over the last six months?” and by choosing one of the following answer options: “stay at home parent; work full-time; work part-time; unemployed; full-time education; retired; something else (please state: …).”

#### 2.3.2. Anthropometry

Standing height was measured without shoes and was recorded to the nearest tenth of a centimetre (i.e., 0.1 cm) using telescopic stadiometers: SECA 213, SECA 214, SECA 217, and SECA 225. Body weight was measured with light clothing and without shoes and recorded to the nearest 0.1 kg. The equipment included electronic weight scales, SECA 813 and SECA 877. Body mass index (BMI) was calculated according to the WHO formula and its reporting followed the WHO classification for adults [1] and the International Obesity Task Force classification for children [18,19].

### 2.4. Data Analysis

All statistical analyses were performed using the Statistical Package for Social Sciences (SPSS Inc., Chicago, IL, USA), version 25.0. The normality of the distribution of continuous variables was tested by the Kolmogorov–Smirnov test. Normally distributed continuous variables are presented as means and standard deviations (SD), while categorical variables are presented as percentages (%).

Between-group differences of continuous variables were tested using either one-way Analysis of Variance (ANOVA) or the independent samples T-test. The significance of the association between categorical variables was examined using the chi-squared (χ^2^) test. Sub-analyses were carried out by economic classification of countries at the time of submission of the study protocol (2014–2015), i.e., “low income” (Bulgaria and Hungary), “high-income under economic crisis” (Greece and Spain), and “high income” (Belgium and Finland). Regarding the characterization of Greece and Spain as counties under economic crisis, this is a definition that was based on historical financial data indicating that both Greece and Spain faced a sovereign debt crisis following the world financial crisis of 2007–2008 [20]. This resulted in a series of reforms and austerity measures that led to recession, loss of income, and a negative impact on both countries’ healthcare systems in the following years, which coincided with the time-period when the Feel4Diabetes study was conducted.

Multivariate logistic regression analyses were carried out to examine associations between childhood obesity (independent variable) and the gender of children, the economic classification of the country they lived in, and the socio-demographic and anthropometric characteristics of their parents at baseline (dependent variables). A parental obesity categorical dependent variable was constructed using the BMI data of the parents. The categories were “no parent with obesity”, when neither parent was obese, “one parent with obesity”, when either the mother or the father was obese, and “both parents with obesity”, when both the father and the mother were obese. All reported *p*-values were two-tailed, and the level of statistical significance was set at *p* < 0.05.

## 3. Results

### 3.1. Weight Status of Participating Children

**Figure 1:**
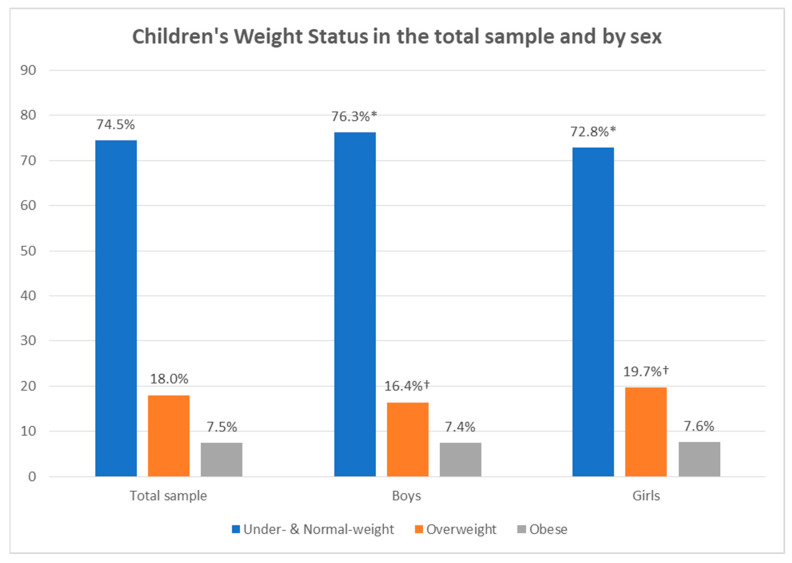
Children’s weight status in the total sample and by sex. *, †: Statistically significant differences (*p* < 0.05) in the pairwise comparison of percentages that share the same superscript symbol based on the χ^2^ test.

Figure 1 presents the weight status of participating children. Three-quarters (74.5%) of participating children in the study either had normal weight or were underweight. Nearly one-fifth (18%) were overweight, and about 1 in 13 children (7.5%) were obese. Significantly more boys either had normal weight or were underweight (76.3%) compared to girls (72.8%); girls were statistically significant more overweight (19.7%) compared to boys (16.4%). No statistically significant difference was observed in the percentage of obese boys (7.4%) compared to obese girls (7.6%).

### 3.2. Parental Socio-Demographic Characteristics and Obesity Status

Table 1 presents the children’s and parental socio-demographic characteristics and the parental obesity status in the total sample and by their country’s economic classification. The mean age of the children was 8.2 (±1.0) years, and there was near-equal representation of both genders (50.6% girls). The study recruited mostly young parents, with 90.4% of mothers and 77.7% of fathers being less than 45 years of age at baseline. Most of the parents had completed high school, at least two years of tertiary education (52% of mothers and 46% of fathers) and were employed full time (6 in 10 mothers and more than four-fifths of fathers). Most mothers were either normal weight or underweight, whereas the majority of the fathers (68.5%) were overweight or obese. When looking at the BMI classification of the parents together, in most families (72%) the parents were not obese, with one in four families (24%) having at least one parent with obesity and one in 26 families (3.8%) having both parents with obesity.

Examining these data by the economic classification of the participating countries revealed statistically significant differences between “low income”, “under economic crisis”, and “high-income” countries. The details are reported in Table 1 (superscripts).

### 3.3. Weight Status of Participating Children by Economic Classification of Country and Parental Socio-Demographic Characteristics

Table 2 presents the weight status of participating children by the economic classification of their country and their parents’ socio-demographic characteristics. In the “low income” countries (Bulgaria and Hungary), 74.5% of participating children in the study either had normal weight or were underweight, 17.4% were overweight, and 8.2% were obese. Countries “under economic crisis” (Greece and Spain) displayed the highest overweight and obesity rates, with 22.7% of the children being overweight and 9.3% being obese. The lowest overweight and obesity rates were seen in the “high income” countries (Belgium and Finland), where 13.4% of children were overweight and 4.2% were obese.

Childhood overweight and obesity rates were higher when either the mother or the father of the child was older; however, the differences were not statistically significant apart from those cases with obese children and those where the mother was older. Higher childhood obesity prevalence was seen when the education of each parent was less than nine years. Childhood obesity prevalence for the lowest education bracket (<9 years) was significantly different versus the highest education bracket (>14 years) and not versus the middle one (9–14 years). Parental occupation and its association with childhood obesity produced mixed results. Highest childhood obesity prevalence was observed when the mother was unemployed and was higher when the father was part-time employed versus unemployed.

### 3.4. Weight Status of Participating Children by Parental Obesity

**Figure 2:**
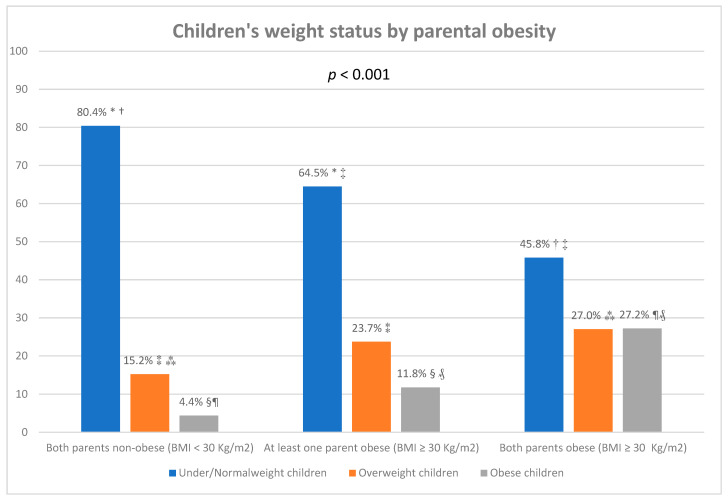
Children’s weight status by parental obesity status. *, †, ‡, ⁑, ⁂, §, ¶, ₰: Statistically significant difference in the comparison of percentages that share the same superscript symbol based on the χ^2^ test (*p* < 0.001).

Figure 2 presents the weight status of participating children by parental obesity. When neither parent was obese, 80.4% of the participating children in the study either had normal weight or were under-weight, 15.2% were overweight, and 4.4% were obese. When at least one parent was obese, 64.5% of children had normal weight or were under-weight, 23.7% were overweight, and 11.8% were obese. When both parents were obese, 45.8% of children had normal weight or were under-weight, 27% were overweight, and 27.2% were obese.

### 3.5. Associations between Childhood Obesity with Country’s Economic Classification, Parental Socio-Demographic Characteristics and Weight Status

Table 3 presents the associations between childhood obesity, the countries’ economic classification, and the socio-demographic characteristics and weight status of participating parents. Odds ratios revealed that children were statistically significantly more likely to be obese if their parents were obese, with children who had at least one parent with obesity having 2.5 times the chance of having obesity themselves (OR: 2.46, 95% CI: 0.32, 0.62) and children whose parents both had obesity being nearly seven times more likely to have obesity themselves (OR: 6.83, 95% CI: 5.15, 9.05). Furthermore, children had higher odds of being obese if they were living in “low-income” countries (OR: 2.11, 95% CI: 1.62, 2.74) and countries “under economic crisis” (OR: 2.48, 95% CI: 1.89, 3.24) as compared to “high-income” countries; if their mother had completed between 9 to 14 years of education (OR: 1.56, 95% CI: 1.26, 1.93); and if their father had completed 9 to 14 (OR: 1.56, 95% CI: 1.26, 1.93) or less than nine years of education (OR: 2.16, 95% CI: 1.54, 3.05) as compared to children whose parents had completed more than 14 years of education.

## 4. Discussion

This study aimed to report the prevalence of childhood obesity in six participating European countries and to examine its associations with family socio-demographic characteristics and the obesity status of parents. Overall, three quarters of participating children either had normal weight or were underweight, with nearly one fifth being overweight and about 1 in 13 children being obese. Girls were more overweight than boys. Studies have reported that girls spend more time being sedentary and less time performing physical activities [21,22,23,24], which could provide one interpretation for this difference between genders; other possible explanations include differences in other energy balance-related behaviours between boys and girls and differences in behaviours related to sleeping and skipping meals [25,26].

More overweight and obese children were seen in the “under economic crisis” countries, i.e., Greece and Spain, compared to both the “low-income” and the “high income” countries of the study, i.e., Bulgaria and Hungary and Belgium and Finland, respectively. The difference in prevalence between Greece and Spain, which were under austerity measures due to recent economic crises, and Belgium and Finland reflect the social and economic diversity between North and South Europe as well as the discrepancy of primary health care services and systems between these regions [27]. The higher overweight and obesity rates seen in the countries “under economic crisis”, i.e., Greece and Spain compared to the “low income” countries, i.e., Bulgaria and Hungary may be due to the fact that only participants residing in lower SES were eligible for recruitment from the former, whereas participants from higher SES were eligible for recruitment from the latter. Lower SES is generally associated with a higher prevalence of unhealthy behaviours [28,29,30] and limited access to primary care services, and people residing in areas with lower socioeconomic status utilise these services less [31,32,33]. Our results agree with previous research which addressed the complex relationship between household income and childhood obesity [34], with higher levels of childhood obesity seen with higher incomes at lower levels of development and with lower incomes at higher levels of development [34].

Childhood overweight and obesity rates were higher when either the father or the mother was middle-aged as compared to cases with younger parent. The fact that younger parents may be more aware of the importance of physical activity and a healthy diet to maintaining healthy body weight could be a mediating factor. Indeed, a higher prevalence of childhood obesity was seen when the education of each parent was less than nine years, with the parents having completed more years of education (>14 years) having children with the lowest prevalence of overweight and obesity. Previously, higher effectiveness of the Feel4Diabetes study has been described among more educated study participants due to the ability of these individuals to better understand and more successfully adopt health messages [10,35,36,37,38]. Additionally, younger parents are generally more physically capable of participating in physical activities with their children, and thus may represent more positive physical activity role models for them [39,40].

Parental occupation and its association with childhood obesity produced mixed results. The highest childhood obesity prevalence was observed when the mother was unemployed, although it was higher when the father was part-time employed versus unemployed. A possible explanation might be that unemployed mothers spend more time at home with the children and provided them with more foods and snacks than necessary to maintain a normal weight. Employment status often correlates with SES, and the latter may be what mediates these outcomes [41].

Childhood obesity was positively and strongly associated with the obesity status of the parents, with one in five children being overweight when neither parent was obese, one in four children being overweight when at least one parent was obese, and one in two children being overweight when both parents were obese. The link between obesity and social connections has been described previously [5]. Previous research has additionally indicated that children whose parents are both obese are ten to twelve times more likely to be obese themselves [42,43], an influence which appears to have both genetic and environmental components. Early childhood weight gain is significantly greater in children with overweight or obese parents [44]. Children of overweight parents perform less physical activity and consume more high-fat and unhealthy foods [45,46]. These associations are not surprising when considering that, especially for young children, parents present their first role models; research has previously reported that children adopt the behaviours of their parents [47,48]. Research has previously highlighted different parenting approaches to improving the eating behaviour of children, including active guidance to promote healthy food consumption and rule-making restrictions on unhealthy food items [49]. The fact that families at risk of developing type 2 diabetes were included in this study may be a reason for the high percentages of overweight parents and children, as being overweight is a strong risk factor for developing type 2 diabetes [50]. Our study further enhances previous findings indicating that traditional risk factors such as parental BMI and socioeconomic factors accurately predict childhood obesity, and in fact are better in this respect than genetic scores [51].

The strengths of this study lie in its large sample size and the robust data collection methods of the Feel4Diabetes study, including the standardised way in which anthropometric measurements were conducted in the different study centres involving centrally-trained research team members, which minimised any inter-observer variability [9]. However, the study has known limitations. First, the self-reporting of part of the collected data is prone to both recall bias and social desirability bias. Second, as Feel4Diabetes was an intervention targeting families via schools as an entry point to recruit participants, the results might therefore not be applicable to single adults, families with no children at all, or those with no primary school children. However, within the context of this study’s target population, the results can be generalised to the whole population of adults/families with children attending primary school, as the participation rate of such families was quite high [9].

## 5. Conclusions and Implications for Future Practice and Research

In conclusion, childhood obesity was associated with parental obesity, SES, and the age, education, and employment status of the parents. These findings underline the factors that need to be addressed by effective childhood obesity prevention programs. Future clinical practice should focus on family obesity prevention programs, as these may be more effective than programs targeting children only, due to the strong association between parental and childhood obesity. The SES of the family should be taken into consideration, as this is reflected in the education and employment status of the parents and can facilitate the implementation of more tailored interventions. Future research should further examine these associations in countries other than the six that were included in this study as well as in more socio-demographically diverse populations in order to facilitate the generalisability of the conclusions.

## Figures and Tables

**Table 1:** Children’s and parents’ socio-demographic characteristics and obesity status in the total sample and by their country’s economic classification *.

		Total Sample	By Economic Classification *			
			Low Income	Under Economic Crisis	High Income	*p*-Value
		(*n* = 12,030)	(*n* = 3281)	(*n* = 3948)	(*n* = 4801)	
Child sex (%)	boy	49.4	48.5	50.0	50.0	0.260
	girl	50.6	51.5	50.0	50.0	
Child age [mean (SD)]		8.2 (1.0)	8.5 (1.0) ^a,b^	7.8 (0.9) ^a,c^	8.3 (1.0) ^b,c^	<0.001
Age of Mother (%)	<45 years old	90.4	93.1 ^a^	85.6 ^a,c^	91.9 ^c^	<0.001
	≥45 years old	9.6	6.9 ^a^	14.4 ^a,c^	8.1 ^c^	
Age of Father (%)	<45 years old	77.7	81.3 ^a^	68.6 ^a,c^	83.3 ^c^	<0.001
	≥45 years old	22.3	18.7 ^a^	31.4 ^a,c^	16.7 ^c^	
Education of Mother (%) **	<9 years	8.4	11.9 ^a,b^	7.7 ^a,c^	4.1 ^b,c^	<0.001
	9–14 years	35.3	34.3 ^a^	39.2 ^a,c^	32.6 ^c^	
	>14 years	51.9	53.8 ^a^	53.1 ^b^	63.3 ^a,b^	
Education of Father (%) **	<9 years	9.7	10.9 ^b^	11.3 ^c^	6.2 ^b,c^	<0.001
	9–14 years	44.4	48.3 ^a^	37.9 ^a,c^	45.5 ^c^	
	>14 years	46.0	40.8 ^a,b^	50.8 ^a^	48.3 ^b^	
Occupation of Mother (%)	unemployed/other #	29.5	32.1 ^a,b^	35.5 ^a,c^	19.3 ^b,c^	<0.001
	employed full-time	57.5	62.0 ^a^	48.2 ^a,c^	60.9 ^c^	
	employed part-time	13.1	6.0 ^a,b^	16.5 ^a,c^	19.8 ^b,c^	
Occupation of Father (%)	unemployed/other #	14.1	19.2 ^a,b^	11.9 ^a,c^	9.4 ^b,c^	<0.001
	employed full-time	81.5	75.4^a,b^	83.1^a,c^	88.6 ^b,c^	
	employed part-time	4.3	5.4 ^b^	5.0 ^c^	2.0 ^b,c^	
BMI of Mother (%)	<25 kg/m^2^	66.8	70.0 ^a,b^	65.4 ^a^	63.5 ^b^	<0.001
	25–29.9 kg/m^2^	22.3	20.1 ^b^	23.0 ^c^	24.5 ^b,c^	
	≥30 kg/m^2^	11.0	9.8 ^b^	11.6 ^c^	11.9 ^b,c^	
BMI of Father (%)	<25 kg/m^2^	31.5	27.5 ^b^	30.3 ^c^	39.0 ^b,c^	<0.001
	25–29.9 kg/m^2^	47.5	47.0	49.5 ^c^	45.8 ^c^	
	≥30 kg/m^2^	21.0	25.5 ^a,b^	20.1 ^a,c^	15.2 ^b,c^	
Parental BMI classification (%)	Both parents without obesity (BMI < 30 kg/m^2^)	72.1	68.2 ^a,b^	72.9 ^a,c^	77.0 ^b,c^	<0.001
	At least one parent with obesity (BMI ≥ 30 kg/m^2^)	24.1	28.1 ^a,b^	22.9 ^a,c^	19.7 ^b,c^	
	Both parents with obesity (BMI ≥ 30 kg/m^2^)	3.8	3.7	4.2	3.3	

^a,b,c^: Statistically significant difference (*p* < 0.05) in the pairwise comparison of percentages or mean values that share the same superscript symbol within the same column (under-, normal, or overweight) and factor of analysis (e.g., age of mother) based on the χ^2^ test for categorical variables or one-way Analysis of Variance for continuous variables. The absence of a superscript next to a percentage or mean value indicates that there is no statistically significant difference between countries of different economic classification. *p*-value derived from χ^2^ test. BMI = body mass index, SD = standard deviation. * Countries classified in three economic brackets as “low income” (Bulgaria and Hungary), “under economic crisis” (at the time the data were collected—Greece and Spain), “high income” (Belgium and Finland). ** Having completed less than 9, 9 to 14, or more than 9 years of education. # Never employed, or previously employed, or retired, etc.

**Table 2:** Weight status of participating children by economic classification of country and parental socio-demographic characteristics.

	Children’s Weight Status		
	Under- & Normal Weight	Overweight	Obesity
Country economic classification *	%	%	%
Low Income	74.5 ^a,b^	17.4 ^a,b^	8.2 ^a^
Under economic crisis	68.0 ^a,c^	22.7 ^a,c^	9.3 ^b^
High income	82.4 ^b,c^	13.4 ^b,c^	4.2 ^a,b^
*p*-value	<0.001		
Age of mother			
<45 years old	75.0 ^a^	17.8	7.2 ^a^
≥45 years old	72.1 ^a^	18.8	9.1 ^a^
*p*-value	0.037		
Age of father			
<45 years old	75.3	17.7	7.0
≥45 years old	73.7	19.2	7.1
*p*-value	0.231		
Education of mother **			
<9 years	71.4 ^a^	17.9	10.7 ^a^
9–14 years	69.9 ^b^	20.0 ^a^	10.0 ^b^
>14 years	78.6 ^a,b^	16.3 ^a^	5.1 ^a,b^
*p*-value	<0.001		
Education of father **			
<9 years	69.5 ^a^	18.5	11 ^a,b^
9–14 years	72.1 ^b^	19.4 ^a^	8.4 ^a,c^
>14 years	79.6 ^a,b^	15.9 ^a^	4.5 ^b,c^
*p*-value	<0.001		
Occupation of mother			
unemployed/other #	71.6 ^a,b^	19.4 ^a^	9.1 ^a,b^
employed full-time	75.7 ^a^	17.4	6.8 ^a^
employed part-time	78 ^b^	16.2 ^a^	5.8 ^b^
*p*-value	<0.001		
Occupation of father			
unemployed/other #	73.4	18.5	8.1
employed full-time	75.8	17.6	6.6 ^a^
employed part-time	70.9	18.7	10.4 ^a^
*p*-value	0.007		

*p*-value derived from χ^2^ test. ^a,b,c^: Statistically significant difference (*p* < 0.05) in the pairwise comparison of percentages that share the same superscript symbol within the same column (under-, normal, or overweight) and factor of analysis (e.g., age of mother) based on the χ^2^ test. The absence of a superscript next to a percentage or mean value indicates that there is no statistically significant difference between groups of different parental and socio-demographic characteristics. * Countries classified in three economic brackets as “low income” (Bulgaria and Hungary), “under economic crisis” (at the time the data were collected—Greece and Spain), “high income” (Belgium and Finland). ** Having completed less than 9, 9 to 14, or more than 9 years of education. # Never employed, or previously employed, or retired, etc.

**Table 3:** Multivariate logistic regression analyses for the association between childhood obesity and country’s economic classification, parental socio-demographic characteristics, and parental obesity.

	Dependent Variable: Childhood Obesity	
Independent Variables	OR	95% CI
Sex		
boys	1.00	
girls	1.01	0.86, 1.20
Country economic classification *		
High income	1.00	
Low Income	**2.11**	**1.62, 2.74**
Under economic crisis	**2.48**	**1.89, 3.24**
Age of mother		
<45 years old	1.00	
≥45 years old	1.17	0.88, 1.55
Education of mother **		
>14 years	1.00	
9–14 years	**1.56**	**1.26, 1.93**
<9 years	1.28	0.88, 1.86
Education of father **		
>14 years	1.00	
9–14 years	**1.63**	**1.31, 2.03**
<9 years	**2.16**	**1.54, 3.05**
Occupation of mother		
unemployed/other #	1.00	
employed full-time	1.07	0.88, 1.31
employed part-time	0.88	0.64, 1.20
Occupation of father		
unemployed/other #	1.00	
employed full-time	1.09	0.85, 1.40
employed part-time	1.25	0.83, 1.89
Parental weight status		
Both parents without obesity (BMI < 30 kg/m^2^)	1.00	
At least one parent with obesity (BMI ≥ 30 kg/m^2^)	**2.49**	**2.07, 2.99**
Both parents with obesity (BMI ≥ 30 kg/m^2^)	**6.83**	**5.15, 9.05**

95% CI = 95% confidence interval; BMI = body mass index; OR = odds ratio. * Countries classified in three economic brackets as “low income” (Bulgaria and Hungary), “under economic crisis” (at the time the data were collected—Greece and Spain), “high income” (Belgium and Finland). ** Having completed less than 9, 9 to 14, or more than 9 years of education. # Never employed, or previously employed, or retired, etc. Values in bold indicate statistically significant OR.

## References

[1] World Health Organisation Obesity and Overweight. https://www.who.int/news-room/fact-sheets/detail/obesity-and-overweight.

[2] World Health Organization The Global Health Observatory. Noncommunicable Diseases. https://www.who.int/data/gho/data/themes/noncommunicable-diseases.

[3] Centers for Disease and Control Prevention Morbidity and Mortality Weekly Report. Body Mass Index and Risk for COVID-19–Related Hospitalization, Intensive Care Unit Admission, Invasive Mechanical Ventilation, and Death—United States, March–December 2020. https://www.cdc.gov/mmwr/volumes/70/wr/mm7010e4.htm?s_cid=mm7010e4_x.

[4] World Obesity COVID-19 and Obesity: The 2021 Atlas. The Cost of Not Addressing the Global Obesity Crisis. March 2021. https://www.worldobesityday.org/assets/downloads/COVID-19-and-Obesity-The-2021-Atlas.pdf.

[5] Christakis N.A., Fowler J.H. (2007). The spread of obesity in a large social network over 32 years. N. Engl. J. Med..

[6] Robertson A., Lobstein T., Knai C. Obesity and Socio-Economic Groups in Europe: Evidence Review and Implications for Action. November 2007. https://ec.europa.eu/health/ph_determinants/life_style/nutrition/documents/ev20081028_rep_en.pdf.

[7] World Health Organisation Regional Office for Europe. New analysis from WHO/Europe Identifies Surprising Trends in Rates of Overweight and Obesity across the Region. https://www.euro.who.int/en/health-topics/noncommunicable-diseases/obesity/news/news/2021/12/new-analysis-from-whoeurope-identifies-surprising-trends-in-rates-of-overweight-and-obesity-across-the-region.

[8] Manios Y., Androutsos O., Lambrinou C.-P., Cardon G., Lindström J., Annemans L., Mateo-Gallego R., De Sabata M.S., Iotova V., Kivelä J. (2018). A school- and community-based intervention to promote healthy lifestyle and prevent type 2 diabetes in vulnerable families across Europe: Design and implementation of the Feel4Diabetes-study. Public Health Nutr..

[9] Van Stappen V., Cardon G., de Craemer M., Mavrogianni C., Usheva N., Kivelä J., Wikström K., De Miquel-Etayo P., González-Gil E.M., Radó A.S. (2021). The effect of a cluster-randomized controlled trial on lifestyle behaviors among families at risk for developing type 2 diabetes across Europe: The Feel4Diabetes-study. Int. J. Behav. Nutr. Phys. Act..

[10] Moschonis G., Karatzi K., Apergi K., Liatis S., Kivelä J., Wikström K., Ayala-Marín A.M., Mateo-Gallego R., Tsochev K., Chakarova N. (2020). Socio-Demographic Characteristics and Body Weight Perceptions of Study Participants Benefitting Most from the Feel4Diabetes Program Based on Their Anthropometric and Glycaemic Profile Changes. Nutrients.

[11] The World Bank World Bank Country and Lending Groups–Country Classification. https://datahelpdesk.worldbank.org/knowledgebase/articles/906519.

[12] Association W.M. (2013). World Medical Association Declaration of Helsinki: Ethical principles for medical research involving human subjects. JAMA.

[13] Androutsos O., Anastasiou C., Lambrinou C.-P., Mavrogianni C., Cardon G., Van Stappen V., Kivelä J., Wikström K., Moreno L.A., Iotova V. (2020). Intra- and inter- observer reliability of anthropometric measurements and blood pressure in primary schoolchildren and adults: The Feel4Diabetes-study. BMC Endocr. Disord..

[14] Lexico Middle Age. https://www.lexico.com/en/definition/middle_age.

[15] Merriam-Webster Middle Age. https://www.merriam-webster.com/dictionary/middle%20age.

[16] Collins Middle Age. https://www.collinsdictionary.com/dictionary/english/middle-age.

[17] European Commision/EACEA/Eurydice, 2018. Compulsory Education in Europe 2018/19. Eurydice–Facts and Figures. Luxemburg: Publications Office of the European Union. https://eacea.ec.europa.eu/national-policies/eurydice/sites/default/files/compulsory_education_2018_19.pdf.

[18] Cole T.J., Bellizzi M.C., Flegal K.M., Dietz W.H. (2000). Establishing a standard definition for child overweight and obesity worldwide: International survey. BMJ.

[19] Cole T.J., Flegal K.M., Nicholls D., Jackson A.A. (2007). Body mass index cut offs to define thinness in children and adolescents: International survey. BMJ.

[20] Frieden J., Walter S. (2017). Understanding the Political Economy of the Eurozone Crisis. Annu. Rev. Political Sci..

[21] Guthold R., Stevens G.A., Riley L.M., Bull F.C. (2020). Global trends in insufficient physical activity among adolescents: A pooled analysis of 298 population-based surveys with 1·6 million participants. Lancet Child. Adolesc Health..

[22] Telford R.M., Telford R.D., Olive L.S., Cochrane T., Davey R. (2016). Why Are Girls Less Physically Active than Boys? Findings from the LOOK Longitudinal Study. PLoS ONE.

[23] Verloigne M., Van Lippevelde W., Maes L., Yildirim M., Chinapaw M., Manios Y., Androutsos O., Kovacs E., Bringolf-Isler B., Brug J. (2012). Levels of physical activity and sedentary time among 10- to 12-year-old boys and girls across 5 European countries using accelerometers: An observational study within the ENERGY-project. Int. J. Behav. Nutr. Phys. Act..

[24] Riddoch C.J., Andersen L.B., Wedderkopp N., Harro M., Klasson-Heggebø L., Sardinha L.B., Cooper A.R., Ekelund U. (2004). Physical activity levels and patterns of 9- and 15-yr-old European children. Med. Sci. Sports Exerc..

[25] Brug J., van Stralen M., Velde S.J.T., Chinapaw M.J.M., De Bourdeaudhuij I., Lien N., Bere E., Maskini V., Singh A.S., Maes L. (2012). Differences in weight status and energy-balance related behaviors among schoolchildren across Europe: The ENERGY-project. PLoS ONE.

[26] Forkert E.C.O., De Moraes A.C.F., Carvalho H.B., Manios Y., Widhalm K., González-Gross M., Gutierrez A., Kafatos A., Censi L., De Henauw S. (2019). Skipping breakfast is associated with adiposity markers especially when sleep time is adequate in adolescents. Sci. Rep..

[27] European Parliament Directorate General for Research. Health care systems in the EU. A Comparative Study. Public Health and Consumer Protection Series. https://www.europarl.europa.eu/workingpapers/saco/pdf/101_en.pdf.

[28] Mohammed S.H., Habtewold T.D., Birhanu M.M., Sissay T.A., Tegegne B.S., Abuzerr S., Esmaillzadeh A. (2019). Neighbourhood socioeconomic status and overweight/obesity: A systematic review and meta-analysis of epidemiological studies. BMJ Open.

[29] Yang Z., Phung H., Hughes A.-M., Sherwood S., Harper E., Kelly P. (2019). Trends in overweight and obesity by socioeconomic status in Year 6 school children, Australian Capital Territory, 2006–2018. BMC Public Health.

[30] Ghosh A., Charlton K.E., Batterham M.J. (2016). Socioeconomic disadvantage and its implications for population health planning of obesity and overweight, using cross-sectional data from general practices from a regional catchment in Australia. BMJ Open.

[31] Siopis G., Jones A., Allman-Farinelli M. (2020). The dietetic workforce distribution geographic atlas provides insight into the inequitable access for dietetic services for people with type 2 diabetes in Australia. Nutr. Diet..

[32] Siopis G., Colagiuri S., Allman-Farinelli M. (2020). People with type 2 diabetes report dietitians, social support and health literacy facilitate their dietary change. J. Nutr. Ed. Behav..

[33] Siopis G., Colagiuri S., Allman-Farinelli M. (2020). Doctors identify regulatory barriers for their patients with type 2 diabetes to access the nutrition expertise of dietitians. Aust. J. Prim. Care..

[34] Broyles S.T., Denstel K.D., Church T.S., Chaput J.-P., Fogelholm M., Hu G., Kuriyan R., Kurpad A., Lambert E.V., for the ISCOLE Research Group (2015). The epidemiological transition and the global childhood obesity epidemic. Int. J. Obes Suppl..

[35] Siren R., Eriksson J.G., Peltonen M., Vanhanen H. (2014). Impact of Health Counselling on Cardiovascular Disease Risk in Middle Aged Men: Influence of Socioeconomic Status. PLoS ONE.

[36] Kim S.H. (2016). Educational attainment moderates the associations of diabetes education with health outcomes. Int. J. Nurs Pract..

[37] Karter A.J., Stevens M.R., Brown A.F., Duru O.K., Gregg E.W., Gary T.L., Beckles G.L., Chien-Wen T., Marrero D.G., Waitzfelder B. (2007). Educational disparities in health behaviors among patients with diabetes: The Translating Research Into Action for Diabetes (TRIAD) Study. BMC Public Health.

[38] Aaby A., Friis K., Christensen B., Rowlands G., Maindal H.T. (2017). Health literacy is associated with health behaviour and self-reported health: A large population-based study in individuals with cardiovascular disease. Eur. J. Prev. Cardiol..

[39] Brouwer S.I., Küpers L.K., Kors L., Sijtsma A., Sauer P.J.J., Renders C.M., Corpeleijn E. (2018). Parental physical activity is associated with objectively measured physical activity in young children in a sex-specific manner: The GECKO Drenthe cohort. BMC Public Health.

[40] Zecevic C.A., Tremblay L., Lovsin T., Michel L. (2010). Parental Influence on Young Children’s Physical Activity. Int. J. Pediatr..

[41] Bartley M., Owen C. (1996). Relation between socioeconomic status, employment, and health during economic change, 1973–1993. BMJ.

[42] Reilly J.J., Armstrong J., Dorosty-Motlagh A.-R., Emmett P., Ness A., Rogers I., Steer C., Sherriff A. (2005). Early life risk factors for obesity in childhood: Cohort study. BMJ.

[43] Whitaker K.L., Jarvis M.J., Beeken R.J., Boniface D., Wardle J. (2010). Comparing maternal and paternal intergenerational transmission of obesity risk in a large population-based sample. Am. J. Clin. Nutr..

[44] Griffiths L.J., Hawkins S.S., Cole T.J., Dezateux C., Millennium Cohort Study Child Health Group (2010). Risk factors for rapid weight gain in preschool children: Findings from a UK-wide prospective study. Int. J. Obes..

[45] Morgan P.J., Okely A.D., Cliff D.P., Jones R.A., Baur L.A. (2008). Correlates of objectively measured physical activity in obese children. Obesity.

[46] Wardle J., Guthrie C., Sanderson S., Birch L., Plomin R. (2001). Food and activity preferences in children of lean and obese parents. Int. J. Obes. Relat. Metab. Disord..

[47] Scaglioni S., De Cosmi V., Ciappolino V., Parazzini F., Brambilla P., Agostoni C. (2018). Factors Influencing Children’s Eating Behaviours. Nutrients.

[48] Savage J.S., Fisher J.O., Birch L.L. (2007). Parental influence on eating behavior: Conception to adolescence. J. Law Med. Ethics..

[49] Yee A.Z., Lwin M.O., Ho S.S. (2017). The influence of parental practices on child promotive and preventive food consumption behaviors: A systematic review and meta-analysis. Int. J. Behav. Nutr. Phys. Act..

[50] Bhupathiraju S.N., Hu F.B. (2016). Epidemiology of Obesity and Diabetes and Their Cardiovascular Complications. Circ. Res..

[51] Morandi A., Meyre D., Lobbens S., Kleinman K., Kaakinen M., Rifas-Shiman S.L., Vatin V., Gaget S., Pouta A., Hartikainen A.L. (2012). Estimation of newborn risk for child or adolescent obesity: Lessons from longitudinal birth cohorts. PLoS ONE.

